# Relative Age Effect on Youth Female Volleyball Players: A Pilot Study on Its Prevalence and Relationship With Anthropometric and Physiological Characteristics

**DOI:** 10.3389/fpsyg.2019.02737

**Published:** 2019-12-03

**Authors:** Sophia D. Papadopoulou, Sousana K. Papadopoulou, Thomas Rosemann, Beat Knechtle, Pantelis T. Nikolaidis

**Affiliations:** ^1^Department of Physical Education and Sport Science, Laboratory of Evaluation of Human Biological Performance, Aristotle University of Thessaloniki, Thessaloniki, Greece; ^2^Department of Nutritional Sciences and Dietetics, International Hellenic University, Thessaloniki, Greece; ^3^Institute of Primary Care, University of Zurich, Zurich, Switzerland; ^4^Exercise Physiology Laboratory, Nikaia, Greece

**Keywords:** birth quarter, body composition, human performance, jumping ability, isometric muscle strength

## Abstract

The relative age effect (RAE) on human performance has been well studied in many sports, especially in soccer; however, little information has been available about the prevalence of RAE in volleyball, and its role on anthropometric and physiological characteristics. The aim of the present study was to examine (a) the prevalence of RAE in selected (i.e., to be considered for the national team) and non-selected youth female volleyball players, and (b) the relationship of birth quarter (BQ) with anthropometric and physiological characteristics. Selected (*n* = 72, age 13.3 ± 0.7 years, weight 62.0 ± 7.2 kg, height 1.72 ± 0.06 m) and non-selected female volleyball players (*n* = 53, age 13.9 ± 1.1 years, weight 56.4 ± 7.3 kg, height 1.66 ± 0.06 m) performed a series of anthropometric and physiological tests. Twenty-six selected participants were born in the first quarter of the year, 19 in the second, 14 in the third, and 13 in the forth. The corresponding frequency by BQ in non-selected participants was 12, 12, 17, and 12. No association was observed between the number of participants and their frequency by BQ neither in the selected (*χ*^2^ = 2.79, *p* = 0.425) nor in the non-selected group (*χ*^2^ = 0.64, *p* = 0.886). Anthropometric and physiological characteristics did not vary by BQ (*p* > 0.05). The absence of RAE in female volleyball players and the similarities of anthropometric and physiological characteristics among BQ might be due to technical-tactical character of this sport. These findings would be of great practical value for coaches and fitness trainers working with young volleyball players.

## Introduction

The relative age effect (RAE) on human performance – i.e., the larger prevalence of athletes born in the first months (e.g., first quarter) of the year (“early born”) compared to their counterparts born in the last months (e.g., last quarter) of the year (“late born”) – has attracted an increased scientific interest during the last three decades considering its relevance for sport performance (Barnsley et al., 1992) and other domains of human performance (Alsaker and Olweus, 1993). This phenomenon indicated a potential advantage of “early born” compared to “late born” athletes (Duarte et al., 2019). So far, most of the research of RAE in sports has been conducted in soccer (Peña-González et al., 2018; Schroepf and Lames, 2018; Yagüe et al., 2018) and focused on the prevalence of RAE analyzing the distribution of births among months of year. On the other hand, less information exists in female volleyball (Okazaki et al., 2011), which has been one of the most popular team sports in women worldwide (Deaner et al., 2012), and – to the best of our knowledge – no study has ever examined the relationship of RAE with anthropometric and physiological characteristics in this sport.

The findings of existing literature on RAE in volleyball have been controversial so far. An absence of RAE has been observed in Dutch volleyball (Van Rossum, 2006), elite Brazilian adult female volleyball players (Parma and Penna, 2018) and Israeli Division 1 (Lidor et al., 2014). On the other hand, RAE has been shown in the top Japanese volleyball league (Nakata and Sakamoto, 2012), elite Brazilian youth female volleyball players (Okazaki et al., 2011) and female United Kingdom school-children 11–18 years volleyball players (Reed et al., 2017). With an exception, where an over-representation of the last quarters of the year for the whole population in recreational volleyball players was found (Larouche et al., 2010), RAE indicated a higher prevalence of “early born” volleyball players especially in the younger age groups suggesting that RAE was attenuating with age in volleyball. This observation was in agreement with findings in soccer, where RAE was less remarkable in the older soccer players compared to their younger counterparts (Brustio et al., 2018).

Considering the above-mentioned literature on volleyball with some studies observing RAE (Okazaki et al., 2011; Nakata and Sakamoto, 2012; Reed et al., 2017) and others not (Van Rossum, 2006; Lidor et al., 2014; Parma and Penna, 2018), it was suggested that further research on the prevalence of RAE in this sport was needed. Such information would be of great practical interest for volleyball practitioners and policy makers, since an observation of disproportionally high number of “early born” volleyball players would indicate a bias against their “late born” counterparts increasing the risk of drop-outs. This topic was particularly important in adolescence, which was a crucial period for the adherence in sports (Soares et al., 2019). Furthermore, it would be of great practical importance to examine the relationship of RAE with anthropometric and physiological characteristics related to performance in female volleyball players. It has been shown that female volleyball players of high performance level were taller, jumped higher and had larger handgrip muscle strength than their counterparts of lower performance level (Nikolaidis et al., 2015). Also, more successful female volleyball players were taller, lighter and scored higher in standing broad jump and medicine ball throw than their less successful counterparts (Milić et al., 2017). Thus, it would be interesting to examine whether “early born” volleyball players would exhibit superior anthropometric and physiological characteristics compared to “late born.” Maturation has been considered previously as a confounding factor of RAE (Peña-González et al., 2018), since early maturers exhibited superior performance than late maturers (Cripps et al., 2016). Therefore, the aim of the present study was to examine (a) the prevalence of RAE in selected and non-selected female volleyball players, and (b) the relationship of RAE with anthropometric and physiological characteristics. Based on relevant research in soccer (Buchheit et al., 2014; De Oliveira Matta et al., 2015), it was hypothesized that RAE would be observed in volleyball players, “early born” would have superior anthropometric and physiological characteristics than “late born” volleyball players, and RAE would have larger magnitude in selected than non-selected volleyball players. For the purpose of this study, “selected” referred to volleyball players who were selected by national team coaches to be considered for the national team of their age group.

## Materials and Methods

A cross-sectional study design was used in the present research. Birth quarter (BQ), i.e., the quarter of birth, was defined as the independent variable, whereas anthropometric and physiological characteristics were designated as dependent variables. Selected (*n* = 72, age 13.3 ± 0.7 years, weight 62.0 ± 7.2 kg, height 1.72 ± 0.06 m) and non-selected female volleyball players (*n* = 53, age 13.9 ± 1.1 years, weight 56.4 ± 7.3 kg, height 1.66 ± 0.06 m) participated in the present study. Selected volleyball players competed in volleyball clubs in Athens. Non-selected volleyball players were members of two youth academies of competitive volleyball clubs from Athens (Greece). All procedures were in accordance with the Declaration of Helsinki as revised in 2008 and approved by the local Institutional Review Board. Participants’ parents or guardians provided informed consent prior to exercise testing session. All participants played volleyball at least three years before the study, had three to four training sessions and one official match weekly.

The testing session was carried out during competitive period in indoor volleyball court. It lasted 90 min, and included a supervised warm-up (10 min submaximal running and 5 min stretching exercises) and the following tests in the specific order: weight, height, skinfolds’ thickness, sit-and-reach test (SAR), Abalakov jump (AJ), four tests of isometric muscle strength (right and left handgrip, lifting with extended and flexed knees) and 20 m endurance shuttle run test (SRT). Two trials were performed for SAR, AJ, and right and left handgrip test, and the best score was recorded for each of these tests. 1 min break was provided between trials and 5 min break among tests. Although this physical fitness test battery was not sport-specific, e.g., it did not include tests corresponding to movements usually performed in volleyball, the selected tests have been used widely due to their ability to discriminate volleyball players by performance level and playing position (Nikolaidis et al., 2015; Sattler et al., 2015; Brazo-Sayavera et al., 2017; Milić et al., 2017; Paz et al., 2017).

An electronic scale (HD-351 Tanita, Arlington Heights, IL, United States) and a stadiometer (SECA, Leicester, United Kingdom) were used to measure weight and height, respectively. Body mass index (BMI) was calculated using the formula “weight (kg)/height (m)^2^.” Body fat percentage (BF%) was predicted using the sum of ten skinfolds’ thickness (cheek, wattle, chest I, triceps, subscapular, abdominal, chest II, suprailiac, thigh, and calf; skinfold caliper Harpenden, West Sussex, United Kingdom) (Parizkova, 1978). The difference from the age at peak height velocity (Δaphv) was evaluated only in the selected group – because sitting height was measured only in this group – and was used as a measure of maturation (Mirwald et al., 2002). A parameter that was evaluated only in the selected Opto-jump system (Microgate Engineering, Bolzano, Italy) was used to measure AJ, i.e., jumping ability of single vertical jump with countermovement and arm-swing (Bosco et al., 1983). Flexibility was tested by SAR on a box providing 15 cm advantage, i.e., the participant got a 15 cm score when reaching the toes of her feet (Adam et al., 1988). Aerobic capacity was assessed by SRT, a widely used graded exercise test (Adam et al., 1988). Isometric muscle strength was evaluated as the sum of four measures (right and left handgrip test, lifting with extended and flexed knees tests; use of digital handgrip and back-and-leg digital dynamometer; Takei, Tokyo, Japan) and expressed either in absolute (kg) or relative (kg/kg of body weight) values (Heyward and Gibson, 2014).

All variables were expressed using mean and standard deviations. Statistical analyses were carried out on IBM SPSS v.20.0 (SPSS, Chicago, IL, United States) and GraphPad Prism v. 7.0 (GraphPad Software, San Diego, CA, United States). A t test examined differences in all measures between the selected and non-selected group. A chi-square test (χ^2^) examined the association of the number of participants by BQ with expected values. Differences in – adjusted for age – anthropometric and physiological characteristics among BQ groups were examined by one-way multivariate analysis of covariance (MANCOVA). In the case of the selected group, the differences were adjusted for both age and Δaphv. The magnitude of the differences was tested by partial eta square, evaluated as small (0.010 < η*_*p*_^2^* ≤ 0.059), medium (0.059 < η*_*p*_^2^* ≤ 0.138), and large (η*_*p*_*^2^ > 0.138) (Cohen, 1988). The relationship among variables was examined by Pearson’s product moment correlation coefficient (*r*), whose magnitude was interpreted as trivial (*r* < 0.10), small (0.10 ≤ *r* < 0.30), moderate (0.30 ≤ *r* < 0.50), large (0.50 ≤ *r* < 0.70), very large (0.70 ≤ *r* < 0.90), nearly perfect (*r* ≥ 0.90), and perfect (*r* = 1.00) (Batterham and Hopkins, 2006). Significance was set at alpha = 0.05.

## Results

**FIGURE 1:**
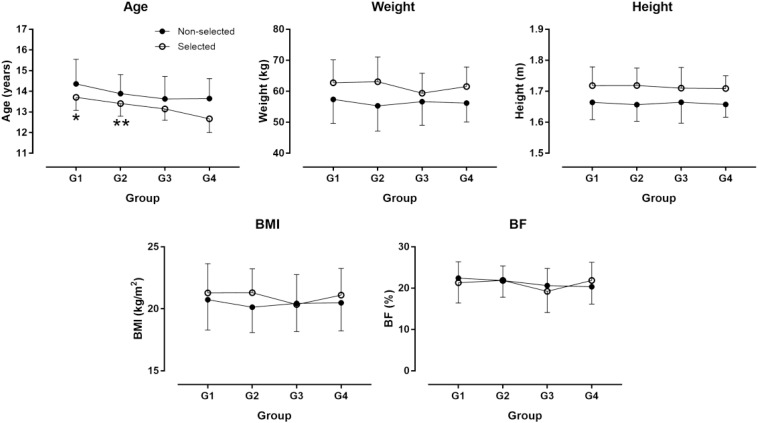
Age and anthropometric characteristics by birth quarter. G1 = born in January, February, and March; G2 = born in April, May, and June; G3 = born in July, August, and September; G4 = born in October, November, and December; BMI = body mass index; BF = body fat percentage. ^∗^G1 older than G3 and G4, ^∗∗^G2 older than G4 in selected participants at *p* < 0.05.

**FIGURE 2:**
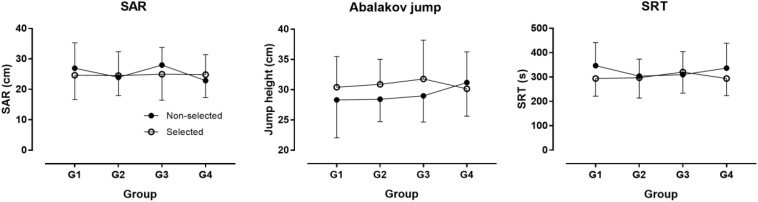
Jumping, flexibility, and aerobic capacity by birth quarter. G1 = born in January, February, and March; G2 = born in April, May, and June; G3 = born in July, August, and September; G4 = born in October, November, and December; SAR = sit-and-reach test; SRT = 20 m endurance shuttle run test. Error bars represent standard deviations.

**FIGURE 3:**
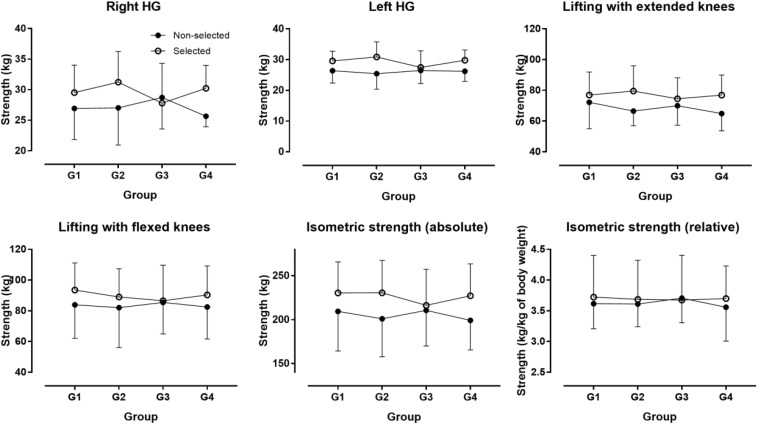
Isometric muscle strength by birth quarter. G1 = born in January, February, and March; G2 = born in April, May, and June; G3 = born in July, August, and September; G4 = born in October, November, and December; “Isometric strength” refers to the sum of the four measures (right and left handgrip strength, lifting with extended and flexed knees. Error bars represent standard deviations.

**FIGURE 4:**
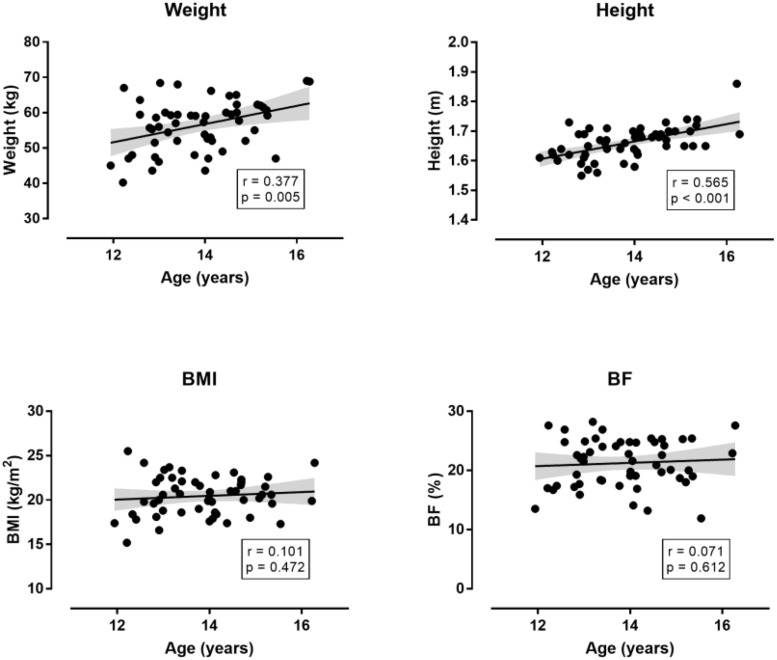
Relationship of anthropometric characteristics with age in the non-selected group (*n* = 53). BMI = body mass index; BF = body fat percentage. The shadow line represents 95% confidence intervals.

**FIGURE 5:**
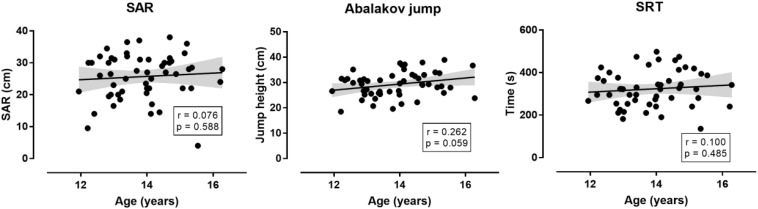
Relationship of jumping, flexibility and aerobic capacity with age in the non-selected group (*n* = 53). SBJ = standing broad jump; SAR = sit-and-reach test; SRT = 20 m endurance shuttle run test. The shadow line represents 95% confidence intervals.

**FIGURE 6:**
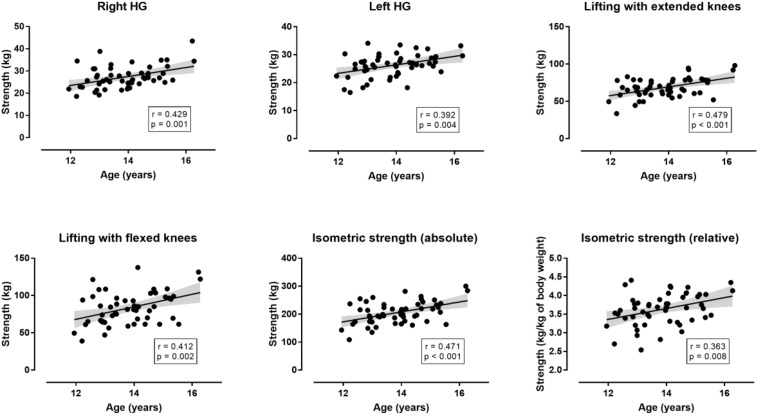
Relationship of isometric muscle strength with age in the non-selected group (*n* = 53). “Isometric strength” refers to the sum of the four measures (right and left handgrip strength, lifting with extended and flexed knees. The shadow line represents 95% confidence intervals.

The descriptive characteristics of participants are presented in Table 1. Twenty-six selected participants were born in the first quarter of the year, 19 in the second, 14 in the third, and 13 in the forth. The corresponding numbers in non-selected participants were 12, 12, 17, and 12. No association was observed between the number of participants and their frequency by BQ neither in the selected (*χ*^2^ = 2.79, *p* = 0.425) nor in the non-selected group (*χ^2^* = 0.64, *p* = 0.886). In the non-selected group, there was no statistically significant difference among BQ groups on the combined dependent variables after controlling for age in the non-selected group [*F*(_36, 104_) = 1.198, *p* = 0.239, Wilks’ Λ = 0.359, η_*p*_^2^ = 0.239]. In the selected group, no statistically significant difference among BQ groups on the combined dependent variables after controlling for age and Δaphv [*F*(_36, 157_) = 0.881, *p* = 0.663, Wilks’ Λ = 0.581, η_*p*_^2^ = 0.165] (Figures 1–3). The relationship of anthropometric and physiological characteristics with age in the non-selected group was shown in Figures 4–6.

**TABLE 1:** Descriptive statistics (mean ± standard deviation) of anthropometric and physiological characteristics of participants.

**Variable**	**Non-selected**	**Selected**
	**(*n* = 53)**	**(*n* = 72)**
Age (years)	13.9 ± 1.1	13.3 ± 0.7^***^
**Anthropometric characteristics**		
Weight (kg)	56.4 ± 7.3	62.0 ± 7.2^***^
Height (m)	1.66 ± 0.06	1.72 ± 0.06^***^
BMI (kg/m^–2^)	20.4 ± 2.2	21.1 ± 2.2
BF (%)	21.2 ± 4.1	21.2 ± 4.5
**Physiological characteristics**		
AJ (cm)	29.2 ± 4.9	30.8 ± 5.0
SAR (cm)	25.7 ± 7.2	24.7 ± 7.4
SRT (min:s)	5:22 ± 1:24	5:00 ± 1:17
**Isometric muscle strength**		
Right HG (kg)	27.2 ± 5.0	29.8 ± 4.5^∗∗^
Left (kg)	26.2 ± 4.1	29.5 ± 4.3^***^
Lifting with extended knees (kg)	68.5 ± 12.9	77.1 ± 14.6^∗∗^
Lifting with flexed knees (kg)	83.7 ± 21.6	90.4 ± 19.0
Isometric strength (kg)	205.6 ± 40.0	227.1 ± 36.6^∗∗^
Isometric strength (kg/kg of body weight)	3.63 ± 0.43	3.70 ± 0.64

*BMI = body mass index; BF = body fat percentage; SAR = sit-and-reach test; SRT = 20 m shuttle run test; HG = handgrip muscle strength; ^∗∗∗^*p* < 0.001, ^∗∗^*p* < 0.01.*

The relationship of anthropometric and physiological characteristics with age and Δaphv in the selected group can be seen in Table 2. Age did not correlate with of the other measures. Δaphv correlated very largely with height, moderately with weight, and with small magnitude with left handgrip muscle strength.

**TABLE 2:** The relationship (Pearson correlation coefficient *r*) of anthropometric and physiological characteristics with age and difference from the age at peak height velocity in the selected group (*n* = 72).

**Variable**	**Age**	**Δaphv**
***Anthropometric characteristics***		
Weight	0.05	0.44^∗∗∗^
Height	0.21	0.72^∗∗∗^
BMI	–0.08	0.02
BF	–0.13	0.05
***Physiological characteristics***		
AJ	0.22	–0.02
SAR	0.03	–0.14
SRT	–0.06	–0.09
***Isometric muscle strength***		
Right HG	0.16	0.21
Left HG	0.11	0.24^∗^
Lifting with extended knees	0.07	0.15
Lifting with flexed knees	0.10	0.17
Absolute isometric strength	0.12	0.21
Relative isometric strength	0.09	–0.10

*BMI = body mass index, BF = body fat percentage, AJ = Abalakov jump, SAR = sit-and-reach test, SRT = 20 m shuttle run test, HG = handgrip muscle strength, Δaphv = difference from the age at peak height velocity. ^∗^*p* < 0.05, ^∗∗∗^*p* < 0.001.*

## Discussion

The main findings of the present study were that, (a) RAE was not observed in selected and non-selected female volleyball players, (b) anthropometric and physiological characteristics did not differ among BQ groups, and (c) the relationship of anthropometric and physiological characteristics with age varied by performance group with stronger correlations observed in the non-selected than in the selected group.

The absence of RAE in the examined sample of female young volleyball players was in agreement with previous studies in volleyball that did not show any difference on the frequency of BQ groups (Van Rossum, 2006; Lidor et al., 2014; Parma and Penna, 2018). An explanation of the absence of RAE in our sample might be that volleyball has been considered a team sport that did not require exceptional demands in physiological characteristics (Lidor and Ziv, 2010a) and was characterized by large variability in these characteristics (Nikolaidis et al., 2012). However, it was acknowledged that other studies conducted on this sport (Okazaki et al., 2011; Nakata and Sakamoto, 2012; Reed et al., 2017) observed RAE highlighting the overall conflicting findings in research on volleyball and addressing the need of further research on this topic. Based on the findings of the present study, it might be assumed that sport and human performances without high demands in physiological characteristics – e.g., aerobic capacity, muscle strength and speed – would attenuate the occurrence of RAE.

The absence of RAE in the present study was in disagreement with the existed literature on team sports with high demands in physiological characteristics. For instance, most studies (Korgaokar et al., 2018; Peña-González et al., 2018; Raᵭa et al., 2018; Schroepf and Lames, 2018; Yagüe et al., 2018; Marques et al., 2019) in soccer have observed an occurrence of RAE, where most soccer players were born in the first quarter or half of the year. Moreover, it has been shown that the number of soccer players born in January would be twice the number of those born in December in the top five European leagues (Raᵭa et al., 2018). An occurrence of RAE would have implications for talent identification and soccer players’ selection and would require action to balance the chances of success for players born in the end of a year (Yagüe et al., 2018). On the contrary, such a bias in talent identification and players’ selection should not be a concern in volleyball.

The similar anthropometric and physiological characteristics among BQ were in agreement with the absence of RAE in the present study. This relationship has been examined previously in soccer, where some studies supported an association between BQ and these characteristics, i.e., “early born” showed superior characteristics than “late born” (Pedretti and Seabra, 2015; Altimari et al., 2018), whereas other studies did not observe differences (De Oliveira Matta et al., 2015; Junior et al., 2015; Lovell et al., 2015; Skorski et al., 2016; Peña-González et al., 2018). An explanation of the similar anthropometric and physiological characteristics among BQ might be the role of maturation as a covariate (Lovell et al., 2015; Peña-González et al., 2018).

With regards to the role of chronological age, the findings in the non-selected group showed that weight, height and isometric muscle strength increased with age, whereas BMI, BF and the other physiological characteristics did not. On the contrary, no relationship was observed between age and these characteristics in the selected group. Considering the adolescence as a period with large changes in the characteristics of volleyball players (Lidor and Ziv, 2010b), the variation in the abovementioned relationship by performance level might be partially attributed to the smaller age range of the selected compared to the non-selected group indicating that the former group was more homogeneous than the latter one. In addition, the variation of this relationship when Δaphv – measure of maturation – was considered instead of chronological age, confirmed the important role of maturation during volleyball players’ selection (Melchiorri et al., 2017; Nunes et al., 2019), since height (a major determinant of success in volleyball) correlated very largely with Δaphv.

A limitation of the present study was that it was conducted in young volleyball players and it would be needed caution to generalize the findings in adult volleyball players, as it has been observed in other team sports (e.g., soccer) that the prevalence of RAE might vary by age group (Lovell et al., 2015; Korgaokar et al., 2018). Moreover, the administered fitness batter included tests corresponding to important parameters for volleyball performance (e.g., height and jump ability) (Nikolaidis et al., 2015; Milić et al., 2017); however, future studies should include sport-specific tests to mimic volleyball movements. In addition, it was acknowledged that the adopted methodological approach to evaluate maturation based on a combination of anthropometric characteristics and chronological age (Mirwald et al., 2002) provided only a proxy measure. Although this approach has been used widely in research on maturation and team sports performance (Pion et al., 2015; Rubajczyk et al., 2017; Lovell et al., 2019; Rommers et al., 2019), it would be recommended that future studies use laboratory methods (e.g., Tanner scale, hand-wrist skeleton), too. On the other hand, strength of the study was its novelty as it was the first one to examine differences in anthropometric and physiological characteristics among BQ of volleyball players. These findings would be of both practical and theoretical importance for practitioners and scientists, respectively. From a practical perspective, it would be suggested that RAE should not be a concern of volleyball coaches and fitness trainers, in contrast with soccer where practitioners should manage the selection bias of their athletes due to the prevalence of RAE. Nonetheless, coaches and fitness trainers should monitor BQ of their volleyball players, especially in the context of players’ selection; in case they observed RAE, they should act (e.g., setting individualized fitness goals) to prevent drop-out of potential talents. From a theoretical point of view, the absence of RAE observed in the young volleyball players under examination might imply that human performance not relying on high levels of physical abilities would not be influenced by BQ at young age. Moreover, scientists interested in this topic should examine further the prevalence of RAE and its role on anthropometric and physiological characteristics especially in sports with more technical than physical demands. With regards to the role of performance level, recently it was observed in soccer that RAE was more prevalent in elite than in non-elite academies (Bezuglov et al., 2019). Thus, future studies should be conducted on the variation of RAE by performance level in volleyball using large sample size to verify this trend.

## Conclusion

The absence of RAE in female volleyball players and the similarities of anthropometric and physiological characteristics among BQ might be due to technical-tactical character of this sport. These findings would be of great practical value for coaches and fitness trainers working with young volleyball players.

## Data Availability Statement

The datasets generated for this study are available on request to the corresponding author.

## Ethics Statement

The studies involving human participants were reviewed and approved by the Institutional Review Board, Exercise Physiology Laboratory, Nikaia, Greece. Written informed consent to participate in this study was provided by the participants’ legal guardian/next of kin.

## Author Contributions

SDP and PN: conceptualization, formal analysis, data curation, and visualization. SDP, SKP, and PN: methodology, validation, investigation, and resources. PN: software. SDP, SKP, TR, BK, and PN: writing – original draft preparation and writing – review and editing. SDP, BK, and PN: supervision and project administration.

## Conflict of Interest

The authors declare that the research was conducted in the absence of any commercial or financial relationships that could be construed as a potential conflict of interest.
